# Assessment of Serum Enzymatic Antioxidant Levels in Patients with Recurrent Aphthous Stomatitis: A Case Control Study

**DOI:** 10.1155/2014/340819

**Published:** 2014-12-10

**Authors:** Ishita Gupta, Arvind Shetti, Vaishali Keluskar, Anjana Bagewadi

**Affiliations:** ^1^Department of Oral Medicine & Radiology, Faculty of Dental Sciences, SGT University, Budhera, Gurgaon 122505, India; ^2^Department of Oral Medicine & Radiology, KLE VK Institute of Dental Sciences, Belgaum 590010, India

## Abstract

*Background and Aim.* Recurrent aphthous stomatitis (RAS) is a common oral mucosal disorder characterized by recurrent, painful oral aphthae. Despite extensive research, the exact etiology of RAS remains elusive. Recently oxidant-antioxidant imbalance of the body has been implicated in the pathogenesis of recurrent aphthous stomatitis. Thus, the aim of the study was to evaluate the enzymatic antioxidant levels in patients with recurrent aphthous stomatitis. *Materials and Methods.* The serum levels of superoxide dismutase (SOD), glutathione peroxidase (GPx), and catalase (CAT) were measured in 30 patients with recurrent aphthous stomatitis and compared to the control group, which included 30 healthy subjects. Student's *t*-test was performed for statistical evaluation. *Results.* The mean levels of superoxide dismutase (130.2 ± 15.94 U/mL) and glutathione peroxidase (3527.93 ± 488.32 U/L) were found to be significantly lower in study group as compared to control group (211.9 ± 20.93 U/mL, 8860.93 ± 1105.31 U/L, resp.) (*P* = 0.000) while level of catalase in study group was significantly higher when compared to control group (10981.00 ± 1018.07 U/mL versus 9764.00 ± 1621.19 U/mL) (*P* = 0.000). *Conclusion.* Enzymatic antioxidant system is impaired in recurrent aphthous stomatitis patients and seems to play a crucial role in its pathogenesis.

## 1. Introduction

Recurrent aphthous stomatitis (RAS) is a common oral mucosal disorder. It affects 5–25% of the general population and nearly 50–60% in certain groups like medical and dental students [1, 2]. Minor aphthous ulcers are the most common variety affecting about 80% of patients and are characterized by recurrent attacks of single or multiple painful oral ulcers [3, 4].

The etiology of RAS is multifactorial ranging from immunologic, microbial to local and systemic factors [2]. All these factors implicated in the etiology of RAS finally lead to free radical formation, which disrupts the oxidant-antioxidant balance of the body and causes cellular damage [5]. Protection against the oxidative stress is provided by enzymatic activities such as superoxide dismutase (SOD), catalase (CAT), and glutathione peroxidase (GPx), as well as nonenzymatic antioxidants like vitamins E, C, and A, melatonin, selenium, and uric acid [5].

There have been studies linking increased oxidative stress and decreased antioxidant defense in mucosa of patients with recurrent aphthous ulcers [6–8] but the results have been inconsistent. In view of the lack of sufficient studies pertaining to this issue, the present study was undertaken to evaluate the serum levels of enzymatic antioxidants SOD, GPx, and CAT in patients with recurrent aphthous stomatitis.

## 2. Materials and Methods

The study was approved by the Ethical and Research Committee of KLE VK Institute of Dental Sciences. Subjects of either gender with the chief complaint of oral ulcers in the age group of 15–35 years reporting to the outpatient department of oral medicine and radiology were included in the study.

### 2.1. Selection Criteria

The study group consisted of 30 subjects with clinical presentation of minor recurrent aphthous ulcers. The diagnosis of minor aphthous ulcers was made depending on the major and minor criteria given by Natah et al. [9]. All RAS patients included in the study had recurring episodes of aphthous ulcers at least 3 times a year. The control group included 30 age and sex matched healthy subjects with no history of any episodes of recurrent aphthous ulcers. An informed consent was obtained from every subject and thorough clinical examination was performed. The patients with known history of systemic diseases like diabetes mellitus or hypertension were excluded from the study. The subjects were also excluded if they had a history of tobacco use, had other concurrent oral lesions, or were under a therapeutic regimen of multivitamins, steroids, or other immunomodulatory agents in the past 2 months.

### 2.2. Sample Preparation

After taking aseptic precautionary measures, 5 mL of venous blood was withdrawn from the antecubital vein using sterile disposable syringe and transferred to EDTA containing vial. The blood was centrifuged for 10 minutes at 3000 rpm using REMI R-8C BL Bench top centrifuge and plasma was separated. Biochemical analysis was then carried out for estimation of SOD, GPx, and CAT.

### 2.3. Assay of Enzymatic Antioxidant Activity

The blood sample was analyzed for alterations in the levels of enzymatic antioxidants in recurrent aphthous stomatitis patients as compared to healthy controls. The level of SOD and GPx was estimated using commercially available kits RANSOD and RANSEL, respectively, by Randox Company Ltd., UK. Similar method for estimation of SOD and GPx was utilized by Momen-Beitollahi et al. [10]. The serum level of CAT was estimated using Beutler's E. method as applied by Çimen et al. [7].

### 2.4. Statistical Analysis

All values were expressed as mean ± SD. Student's *t*-test was used to analyze the significance between parameters. The *P* value of less than 0.05 was considered to be statistically significant.

## 3. Results

### 3.1. Demographics

The mean ± SD age of subjects in study and control group was 25.76 ± 4.88 years (range 19–35 years) and 25.13 ± 2.80 years (range 19–32 years), respectively (*P* > 0.05). Both the study and control groups comprised 17 males and 13 females. Hence, the age and gender of the subjects in the study and control group were matched in the present study. It was observed that 16 of the patients (53.33%) with recurrent aphthous ulcers in the present study were dental students.

### 3.2. Antioxidant Levels

Comparison of enzymatic antioxidants in the serum between the study and control group is shown in Table 1. The levels of SOD and GPx were found to be significantly decreased in study group as compared to control group (*P* < 0.001) while the level of CAT in the study group was significantly higher as compared to the control group (*P* < 0.001).

## 4. Discussion

The imbalance between oxidants and antioxidants is responsible for causing a range of inflammatory oral disorders varying from infections and immunologic diseases to lethal cancers [7]. One manifestation of this imbalance is recurrent aphthous ulcers in oral cavity. Histological examination of RAS reveals infiltration of immune system defense cells and the killer activity of these cells increases the concentration of free radicals. Free radicals disrupt the cellular functions and damage fatty acids, proteins, and DNA by causing peroxidation. These free radicals are neutralized by antioxidants and the amount of each antioxidant response has been used to indirectly evaluate free radical activity [10]. In spite of a great deal of effort spent on this subject, there are very few studies in the literature investigating the possible relation between recurrent aphthous stomatitis and oxidant/antioxidant pathway.

The present study was undertaken to evaluate the serum enzymatic antioxidant levels in patients with recurrent aphthous stomatitis and healthy controls. In the study group, 20 patients (66.67%) were between 20 and 30 years. Similar findings have been reported in the literature by Natah et al. and Scully et al. who stated that the peak age of onset for recurrent aphthous stomatitis is the second decade [9, 11]. There were a greater number of males (56.67%) as compared to females in this study which is contrary to the studies in literature which have described a female predominance [9, 12]. However, it is in accordance with the findings of Bagan et al. who found a prevalence of more than 50% in men [13].

More than 50% of the patients were dental students which was consistent with the findings in various epidemiologic studies [1, 2, 12] by Scully et al., Ship, and Miller et al. who reported that, in certain groups like medical and dental students, the prevalence of RAS can reach as high as 50–60%. The reason for the increased prevalence in professional students is thought to be the stress associated with the pressure of academics demonstrating that stress plays a major role in precipitating aphthous ulcers.

The level of SOD was found to be significantly lower in the study group with a “*P*” value of zero (*P* < 0.05) when compared to control group. Our results were in accordance with the studies carried out by Karincaoglu et al. [8], Huang et al. [14], Momen-Beitollahi et al. [10], and Saxena [15] who all found that SOD levels were significantly lowered in RAS patients as compared to controls. However, our results were contrary to the findings of Gunduz et al. and Altinyazar et al. who found that activity of SOD increased in RAS patients [16, 17]. The level of GPx was also found to be significantly lower in study group with a “*P*” value of zero (*P* < 0.05) as compared to control group. These results were in agreement with the findings of Çimen et al. [7] and Arikan et al. [18] but were in contrast to the findings by Karincaoglu et al. [8] and Saxena [15] who in their studies found significantly increased levels of GPx in RAS patients. CAT activity was found to be significantly higher in study group with a “*P*” value of zero (*P* < 0.05) when compared to control group. This finding was consistent with that of Altinyazar et al. who found significantly higher activity of CAT in RAS patients [17]. However, previous studies by Çimen et al. [7], Karincaoglu et al. [8], and Saxena [15] showed a decline in the level of CAT in RAS patients.

SOD is an enzyme that catalyzes the dismutation of two superoxide anions into hydrogen peroxide and molecular oxygen [10]. The decreased level of SOD activity noted in the present study may be due to its high utilization to defend the cells from the injurious effects of superoxide radicals. GPx is the major antioxidant which gets rid of hydrogen peroxide produced during the dismutation of superoxide radicals by consuming reduced glutathione. As GPx eliminates the excess hydrogen peroxide, the consumption of reduced glutathione also increases. Lack or deficiency of glutathione leads to a decrease in the plasma level of GPX in recurrent aphthous stomatitis patients [8]. CAT is recognized to be secondary antioxidant enzyme in peroxidative defense. It hydrolyzes hydrogen peroxide into water and oxygen. The increase noted in the level of CAT could be attributed to feedback effect of hydrogen peroxide on mRNA expression [8, 15].

Çimen et al. [7] in their study found no significant differences in SOD activity in patients with RAS as compared to healthy controls. Momen-Beitollahi et al. [10] reported no significant differences in the levels of CAT and GPx in RAS patients when compared to control subjects. Another study by Gunduz et al. [16] concluded that CAT levels in RAS group and control group did not differ significantly. The relative controversies among different studies in the literature regarding the enzymatic antioxidants may be associated with several factors like sample size variations, application of various methods, and genetic divergence of each population. In addition, the composition and antioxidant capacity may vary with dietary and nutrient deficiency.

The variation in the levels of enzymatic antioxidants in the plasma of RAS patients observed in the present study indicates the oxidant nature of the disease and emphasizes the involvement of oxidants and antioxidants in the pathogenesis of recurrent aphthous stomatitis. The number of new disorders where antioxidant enzyme analysis is proving useful is growing rapidly and it is expected that these analyses will greatly aid as biomarkers in future diagnosis and management. Current research reveals the different potential applications of antioxidant and free radical manipulations in prevention or control of disease. Adjuncts like zinc supplements can be utilized as it decreases the oxidative stress and also increases the levels of superoxide dismutase [19, 20]. Newer and future approaches include gene therapy to produce more antioxidants in the body, genetically engineered plant products with higher levels of antioxidants, synthetic antioxidant enzymes (SOD mimics), novel biomolecules, and the use of functional foods enriched with antioxidants [21].

## 5. Conclusion

The impairment of the enzymatic antioxidant system represents one route of pathogenesis for ulcerative lesions like recurrent aphthous stomatitis and emphasizes that oxidant-antioxidant imbalance plays a crucial role in the inflammatory reactions observed in recurrent aphthous stomatitis. Also evaluation of antioxidant status of patients suffering from recurrent aphthous stomatitis is among the potential determinants of susceptibility to this disorder. Supplementation with antioxidants in patients with RAS may strengthen the blood antioxidant defense and provide increased beneficial therapeutic effectiveness in aphthous ulcers. However, further prospective studies with larger sample size are required in this direction. The results may be very useful in identifying new treatments or preventive options using antioxidant supplementation or nutritional prescriptions that might elevate antioxidant levels in recurrent aphthous stomatitis patients.

## Figures and Tables

**Table 1:** Comparison of enzymatic antioxidant levels in study group and control group.

Group	Superoxide dismutase (U/mL)	Glutathione peroxidase (U/L)	Catalase (U/mL)
Study group (*n* = 30)	130.2 ± 15.94	3527.93 ± 488.32	10981.00 ± 1018.07
Control group (*n* = 30)	211.9 ± 20.93	8860.93 ± 1105.31	9764.00 ± 1621.19
*P*value^*^	<0.001	<0.001	<0.001

^*^ *P* value < 0.005 considered statistically significant.

Values expressed as mean ± standard deviation.
